# The Anatomy of the Large American Fluke (*Fasciola Magna*)

**Published:** 1894-11

**Authors:** Charles Wardell Stiles

**Affiliations:** Zoȯlogist, Bureau of Animal Industry


### THE ANATOMY OF THE LARGE AMERICAN FLUKE (*FASCIOLA MAGNA*), AND A COMPARISON WITH OTHER SPECIES OF THE GENUS FASCIOLA, S.ST.

By Chas. Wardell Stiles, Ph.D.,

*Zoölogist, Bureau of Animal Industry.*

### CONTAINING ALSO A LIST OF THE CHIEF EPIZOÖTICS OF FASCIOLIASIS (DISTOMATOSIS) AND A BIBLIOGRAPHY OF *FASCIOLA HEPATICA*.

By Albert Hassall, M.R.C.V.S.

(Continued from page 313.)

### Bibliography of F. hepatica.

BY ALBERT HASSALL.

Articles the date of which are included in parentheses ( ) are taken upon the authority of other writers. I have personally checked the remaining.

1500-1599.

Gabucinus, Hieronimus. (Fanensis)—
'97.—Commentarius de lumbricis alvum occupantibus et eorum cura. Lugd. Batav.

Gemma, Cornelius—
('75.)—De Naturæ divinis characterismis. Antuerpiæ, t II. lib. II. Cap. II. p. 40.

1600-1699.

Faber, Joh.—
('70.)—Nota in Rechum fol. 610 in G. J. Sachs, Scholion zu Th. Bartholini sanguis verminosus. Lips. Obs. 50 page 147.

Frommani, Joh.—
('75 or '76.)—Obs de verminoso in ovibus et juvencis reperto hepate ; in Ephem. (Misc.) Nat. Cur. dec. 1. an. 7. pp. 249-255.
('76.)—Obs. de salubrit. Carn. animal. Verm. laborant ; Ephem. nat. Cur. pp. 255-262.

Heide, Ant. de—
('86-'88.)—Vermes in hepate ovillo. Ejus experimentis. Amst. pp. 46-47.

Pecquet—
('68.)—Journal des Savants. Paris.

REDI, F.—
'84.—Osserv. di Francesco Redi. Accademico della crusca intorno agli Animali viventi che si trovano negli Anim. viv. in Firenze, p. 133.
WEPFER, J. J.—
('88.) —Ventriculi tumor verminosus cum folliculo; Misc. curios. s. Ephem. Med. Phys. Germ. Academie Imp Leopold Nat. Cur. Dec. II. Ann VII. Anni 1688. Norimb. 1689. Obs. XVI. pp. 26-35 v. p. 31.
WILLIUS, J. Valentin—
('74-'75.)—Collect. Acad. part. Étrang. t. VII. et Act. de Copenhague.

1700-1799.

ANDRY, Nicholas—
('00).—Traité de la génération des vers dans le corps de l'homme; de la nature et des espéces de cette maladie. Avec 5 pl. in 12. Paris.
'01.—An account of the breeding of worms in human bodies: their nature, and several sorts; their effects, symptoms and prognostics. With the true means to avoid them, and med'cines to cure them. London, pp. 121-122. 4 pl.
('08.)—id. (French edition.)
('14.)—id. Amsterdam.
('41.)—id. 2 vols. Paris. t. I. p. 62 et 105.
BILHUBER—
('91.)—Die Egelkrankheit. Ludwigsburg.
BLOCH, Marcus Elieser—
'82.—Abhandlung von der Erzengung der Eingeweidewürmer und den Mitteln wieder dieselben. Berlin. pp. 5-6. Tab. 1. figs. 3-4. (*Fasciola hepatica.*)
BORLASE—
('58.)—Nat. Hist. of Cornwall t. 20, f. 10. Oxford.
BRUGUIERE—
'91.—Tableau Encyclopèdique et methodique des trois Règnes de la nature. Paris. pl. 79, figs. 1-8.
CAMPER, Petr.—
('62.)—De Fasciola hepatica ovium ejusque origine, ad calcem agriculturæ Novæ. Belgicæ editæ Tom. II, p. 304, et taf. IV.
CHABERT—
('91.)—De la pourriture des bêtes à laine; Instr. et observ. sur les mal. des an. dom., nouv. édit., Paris, aniii p 152.
CLERICI, Dan.—
('15.)—Historia naturalis et medica latorum Lumbricorum, in tra hominem et alia animalia nascentium, ex variis auctoribus et propriis observationibus. Accessit, horum occasione, de ceteris quoque hominum vermibus, tum in omnium origine tandemque de remediis quibus pelli possint, disquisitio. Cum variis fig. (in tabl aen. 14) 4 Genevæ, 456 pgs.
('21.)—A natural and medical history of Worms bred in the bodies of men and other animals. With 3 pl. in 8. London. (436 pgs.)
CUVIER, G.—
'98.—*Fasciola hepatica;* Tableau élémentaire de l'histoire Naturelle des Animaux. Paris, An. 6. p. 633-634.
DAUBENTON—
('50-'52.)—Allgemeine Historie der Natur nach allen ihren besondern Theilen abgehandelt; nebst einer Beschreibung der Naturalienkammer des Königs von Frankreich. Hamburg & Leipsig 1-8 Theil.

DESMARS—
('62.) -Mémoires Boulogne.
ELLIS—
('49.)—Shepherd's Sure Guide.
FABRICIUS, Otto—
('80.)—Fauna Grönlandica Systematice sistens animalia Grönlandiæ occidentalis hactenus indagata, etc. Havn. et Lipsiæ. 452 pages. 8°. 1. Tav. p. 327-328.
LA FOSSE—
('72-'74-'79.)—Cours d' Hippiatrique. Paris, 1772. p. 157. *Ascaris vermicularis, Fasciolæ hepaticæ.* Phys. oekon. Bibl. IV. 1774, p. 320. 1779. p. 139.
GOEZE, J. A. E.—
'82.—Versuch einer Naturgeschichte der Eingeweidewürmer thierischer Körper. Blankenburg pp. 169-172. tab. XIV. fig. 1. (*Fasciola hepatica.*)
KULMUS, Joh Ad.—
('21.)—Breslauer Sammlungen, p. 596.
LEEUWENHOEK, Antonium Van.—
('04-'05.)—Part of a letter from Anthony van Leeuwenhoek concerning worms observed in sheep livers and pasture grounds; Philosoph. Transact. Vol. XXIV. for the years 1704, No. 289, pp. 1522-1527. 1705, p. 1522.
'15.—Observatio de animalculis in ovino, aliorumque animantium hepate detectis, Lugduni Batavorum. pp. 1-33, 14 figs.
LINNÉ, Carolus—
'58.—*Fasciola hepatica;* Systema naturæ. Holmiæ. (Ed. x) p. 648-649. Tom. 1.
'60.—*F. hepatica;* Systema naturæ—Halæ Magdeburgicæ, 1760. (From Edit. x by Johannes Joachimus, Langius.)
('61.)—Faun. Suec. Edit. II. p. 505, N. 2075.
'66.—Systema Naturæ. Ed. XII. p 1077. No. 1.
'92.—*F. hepatica;* Systema Naturæ. p. 3053–3054. (Edit. XIII).
MILLS—
('76.)—Treatise on Cattle.
MULLFR, O. F.—
('73.) Vermium terrestrium et fluviatilium seu animalium infusoriorum helminthicorum et testaceorum, non marinorum succincta historia. 2 Vols. 1773–1774. Havn. et Lips.
('76.)—Zoologiæ danicæ prodromus seu animalium Daniæ & Norvegiæ indigenarum characteres, Nomina, etc. Hafn. 8.
('82.)—Naturf. XVIII. St. 34 Tab. III. 11. (ad dextrum.) Halle.
MULLER, Philipp. Ludwig. Statius—
'75.—Des Ritters Carl von Linne Königlich Schwedischen Leibarztes Vollständiges Natursystem VI. Bd. 1. Nürnberg. pp 42–43. (Fasciola hepatica.)
PALLAS. Petr. Sim —
('60.)—Diss. inaug. de infestis viv. intr. viv. 4 Lugduni Batav. (62 pgs.)
'68.—Dissertatio de infestis viventibus intra viventia, quam pro gradu Doctoratus Eruditorum examini submisit. Rotterdam. pp. 269–271.
REDI—
'29.—Opusculorum Pars tertia, sive de animalculis vivis quæ in corporibus animalium vivorum reperiuntur observationes. III Vol. Cum 31. 28 et 26 Tab. aen. 12. Lugduni-Batavorum.

ROMBERG, W.—
('06.) —De verme in hepate; Ac. Caes. Leop. Nat. Cur. Ephem. Dec III. Ann. 9–10 Norimb. 1706 (1701–2). 69–70.
ROZIER, Abbé.—
('74.)—Pourriture du monton; Dictionnaire, Lyons.
RUDOLPHI. C. A.—
('93.)—Observationes circa vermes intestinales Gryphiswald.
RUYSCH, Frid —
'37.—Dilucidatio valvularum, p. 23 obs. XVIII. Cap. III; Opera omnia anatomico-medico-chirurgica. Amstelod. Tom. I.
SCHÆFFER, J. Christ.—
('53.)—Egelschnecken in den Lebern der Schafe und die von diesen Würmern entstehende Schafkrankheit. Regensburg.
Schaeffer—
('26.)—Breslauer Sammlungen, p. 57.
SCHRANK, F. v. P.—
('88.)—Verzeichniss der bisher hinlänglich bekannten Eingeweidewürmer, nebst einer Abhandlung über ihre Anverwandschaften. München. 17. n. 56.
SWEITEN, Van.—
('58.)—Comment. in Aphorismos-Paris, t. III. p. 89.

1800–1894.

Adenot—
('63.)—Journal de méd vétér., Lyon p. 112.
ANACKER—
'85.—Distomatosis hepatica; Art. in Koch's Encyclopädie d. gesammt. Thierheilkunde. Wien u. Leipzig. Bd. II. pp. 380–382.
(ANONYMOUS)—
'36.—Editorial; The Veterinarian. Vol. IX. Rot. pp. 227–228.
'36.—The Rot in Two Sheep; The Veterinarian. Vol. IX. p. 539–540. Transl. from Journ. d. Sc. Zoöiatriques Mai (1836).
'37.—Rules for the Prevention of Rot in Sheep; The Veterinarian. Vol. X. pp. 442-444. From Mém de l'ouest. Jan. (1837).
'38.—The Old Reputation of Salt for the Cure of the Rot; The Veterinarian. Vol. XI. p 112. from The Universal Magazine (1748).
'45.—*Distoma hepaticum, Fasciolaria hepatica;* Encyclopædia Metropolitana or Universal Dictionary of Knowledge. London, p. 141. Vol. XVIII.
'63.—Note on the Rot in Sheep; The Veterinarian. Vol. XXXVI. p. 100.
'63.—Note on Rot in Sheep, Cattle & Hares; The Veterinarian. Vol. XXXVI. p. 156–157. (From Bristol Mirror and Scotsman.)
('63.)—Prevalence of Rot in Sheep from Carlisle Journ. Edin. Vet. Rev. 1863.
('63.)—*Distomum hepaticum* in d. Fettleber eines an morbus Brightü verstorbenen Soldaten. in: Preuss Militärärztl. ztg. 3. Jhg. (No. 2.) p. 15.
('80.)—Zeits. f. Mikr. Fleischschau. Berl. 1879–80 I. 171.
'87.—*Distomum hepaticum;* Sidekrift. Veterinar Medicin. (1882). Am. Vet. Rev. 1887. XI. p. 391.
D'ARBOVAL—
'38.—Art. Cachexie; Dict. d. Méd., d. Chir., et d'Hygiéne vét. Paris. T. I. p. 255.

BAILLET, C.—

'66.—Histoire nat. d. Helm ; Extr. du Nour. Dict d. Méd., de Chir. et d'Hyg. vét. pp. 99–104.

'79.—Note sur le developpement de l'embryon dans les œuf de la Douve hépatique; Mém d. l'acad. de Sci. de Toulouse. I, p. 197–215. 2° semestre.

BAIRD, W.—

'53.—Catalogue of Entozoa (Brit. Mus. Cat.), p. 49.

BASS, E.—

'92.—Einige Bemerkungen zur Geschichte der Thierseuchen; Monatshefte f. prak. Thier. IV. H. pp. 93–95.

BELLINGHAM—

('44.)—Ann. & Mag. Nat. Hist., XIII, p. 423.

BÉNION, Ad.—

('74.)—Cachezie ictero-vermineuse ov distomatose; Traité complet d'elevage et des maladies du mouton. Paris, pp. 632–646.

BERT & BLANCHARD—

'85.—Éléments de Zoologie, Paris. pp. 585–586. fig. 539.

BIEHRINGER, J.—

'84.—Beiträge zur Anatomie und Entwickelungsgeschichte der Trematoden; Arbeiten aus dem Zool.—Zoot. Instituts an der universität Würzburg. VII. p. 1.

BIERMER, A.—

('63.)—Distomum hepaticum beim Menschen; Schweiz. Zeits. für Heilk. II. p. 381–395.

('64.)—Review of '63; Gazette Médicale de Paris, Oct. 22.

'65.—*Distoma hepaticum* in man; Rev. in British & Foreign Medico-chirurgical Review. Jan. p. 253. Vol. XXXV.

BLAINVILLE—

('28.)—Dict. Sci. Nat. LVII. p. 585, t. 41. f. 2.

BLANCHARD, E.—

('47.)—Ann. des Sci. Nat. Zool. pp. 279–291. Tab. XI. 1 et 2. (Anat.)

BLANCHARD, R.—

'89.—*Distoma hepaticum;* Traité de Zoologie Méd. Paris, pp. 543–602. figs. 296–310.

'90.—Anim. Paras. introd. par l'eau. Paris, p. 46. figs. 28–33.—Trans. Los Animales Parásitos introducidos por el agua en el Organismo. Londres. 1890. pp. 66–75. figs. 28–33.

'91.—Note sur quelques parasites de l'homme; C. R. d. Soc. Biol. Distoma hepaticum Linné 1767. pp. 604–606.

BLUMENBACH, Fried.—

'21.—*Fasciola hepatica;* Handbuch der Naturgeschichte. Göttingen. p. 441.

BOJANUS, L.—

('21a.)—Isis. p. 170 et 305, pl. 2, fig. 20–23; pl. 4., fig. a, b, c. (*Distoma hepaticum*) (from Dujardin).

('21b.)—Enthelminthica; Oken's Isis, I. (from R. Blanchard).

('21c.)—Nachtrag zu *Distoma hepaticum;* Isis, 1821. II. p. 162–190. Taf. 2. 3.

BOLLINGER—

('89.)—Thierärztl. Mittheil., p. 177.

DE BONIS—

('76.)—Paras. d. Corpo umano, Napoli. p. 155. tav. II. f. 12.

BONVICINI—

('81.)—La veterinaria.

('82.)—Archives vétérinaires. p. 114.

BOSC—

('02.)—Hist. Nat. Vers. i. p. 268.

BOSTRÖM, Eug.—

('83.)—Ueber *Distoma hepaticum* beim Menschen; Deutsches Arch. f. Klin. Medicin XXXIII. p. 557.

BRAUN—

'83.—Die th. Par. d. Menschen. Würzburg. pp. 59-62, fig. 15.

('91.)—Arch. d. Fr. d. Naturg. l. m. 1891. p. 98.

'93.—Vermes.

BREMSER—

'19.—Über lebende Würmer im lebenden Menschen. Wien. pp. 229–233, pl. IV. fig. 11–14.

'24 —Traité des vers intest, pp. 133 et 265–272. tab. 9. f. 1–1 C.

BRERA, V. L.—

'11.—Memorie fisico—mediche sopra i principali vermi del corpo umano vivente e le cosi dette mallatie verminose per servire di supplemento e di continuazione alle lezione. 4° Crema. *Fasciola epatico,* p 92, tab. 1, fig. 24–25.

BRUSAFERRO, D.—

('87.)—Giornale di med. veter. pratica. Turin, 1887. p. 296.

BUDD, George—

('52.)—On diseases of the Liver. London, p. 481.

BUNCK. W.—

('65.)—Œsterreichische Vierteljahrsschrift. p. 33.

BURKE, R. W.—

'86.—*Distoma hepaticum* in the lungs of Animals; The Veterinarian. Vol. LIX, p. 470.

CADÉAC,—

('85.)—Sur un cas de distomatose observé chez une ânesse; Revue Vétérinaire. p. 10.

CARTER, H. V.—

('62.)—Note on *Distoma hepaticum* (from a patient under the care of Mr. Pandoorung); Bombay, Med. & Phys. Soc. Trans. (appendix)

CHABERT, J. X.—

('52.)—Case of *Distoma hepaticum* or liver worm; Boston Med. & Surg. Journ. XLVI. 173 (Cobbold states this is a case of *Tænia saginata.*)

CHESTER, W. L.—

('85.)—A case of Liver Fluke (*F. hepatica*) Army Med. Dep Rep. 1885. London, 1887, XXVII. 359.

DELLE CHIAJE—

('33.)—*Distoma hepaticum;* Comp. di elmint. uman. 2 édit. 11 et. 115. Tab. II 12.

COBBOLD, T. S.—

'61 —Synopsis Distom. p. 3; Linn. Soc. Proc.

('62.)—The Common Liver Entozoon of Cattle; Edin. New Philosoph. Journ. March, 1862.

('62.)—id.; Intellectual observer, 1862. p. 24-33. pl. 1. 115-123.

'64.—Entozoa. p. 147. tab. XI. London.
'73.—The internal parasites of our Domesticated Animals. London. pp. 6-9. fig. 2.
('71.)—On the Fluke Parasites of our food producing Ruminants. Lec. IV. Cantor Series. Journ. Soc. Arts. London.
'79.—*F. hepatica*; Parasites, a treatise on the Entozoa of Man and Animals. Lond. pp. 14-17, 48, 322-332. (fig. 61. after E. Blanchard).
('80.)—On the Rot in Sheep; The Times, April 7, 1880.
('80.)—id.; Zool. Anz. 3 Jhg. p. 257-258.
'82.—Parasitic Diseases; Williams' Principles & Practice of Veterinary Medicine. Edin. 1882. pp. 699-704. pl. VI. fig. 2-8.

COPE, A. C.—
'87.—*Distomum hepaticum*; Veterinarian, Aug. p. 546.—Am. Vet. Rev. 1887. p. 392.

CPEPLIN—
'45.—Wiegmann's Archiv. p. 148.

CURTICE, C.—
'87.—Distoma in liver and lungs of Cattle; Am. Vet. Rev. XI. p. 390.
'90.—*D. hepaticum;* The Animal Parasites of Sheep.—Washington, D. C. pp 16-17. 127-134. Pl. XVI.

CUVIER—
'17.—Le Régne animal distribué d'aprés son organisation. 8°. Paris. Tom. IV. pp. 41-42.
('29.)—Régne Animal III. 263.
'31.—*Distoma hepatica*; *F. hepatica* L.; The Animal Kingdom arranged in conformity with its organization. By the Baron Cuvier. Vol. IV. p. 364. Translated by H. M. Mutrie. New York.

DAVAINE, C.—
'77.—Traité des Entozoaires, Paris. 2nd. Edit. p. LXXV. figs. 36-38.

DELAFOND—
('53.)—Traité sur la pourriture; mém de la soc. d'agric. Paris.
('54.)—id: 2e Édit. Paris.
('59.)—Rapport à la société centr. d'agr.; Rec. de méd. vét.

DELORME—
('61.)—Journ. de méd. vét., Lyon; p. 241.

DEWITZ. J.—
'92.—*Distomum hepaticum*; Eingeweidewürmer der Haussängetiere. Berlin. pp. 117-125. figs. 62, 66, 68, 70, 75, 77 & 78.

DIDRY—
('32.)—De la cachexie aqueuse on hydroposie des bêtes á grosses cornes; Rec. de Méd. Vétér. prat. p. 139.

DIESING, C. M.—
'50.—Syst. Helm. I, p. 332.
'58.—Wiener Sitzb. XXXII. p. 331.
'59.—Wiener Sitzb. XXXV. p. 427.

DROSSE—
('56.)—Preuss. mittheilungen.

DUGUID & COPE—
'87.—The Veterinarian. Lx. p. 385.

DUJARDIN, F.—

'45.—*Distoma* (Cladocoelium) *hepaticum*; Histoire Naturelle des Helminthes. p. 389. Paris.

DUVAL—

('42.)—Note sur un cas de présence du distome hépatique (douve du foie) dans la veine-porte chez l'homme; Gaz. Méd. de Paris., 1842, 2. s., X, 769-772.

ERCOLANI, G. B.—

'81a.—Sull' ovulatzione dei Distomi epatico e lanceolato delle pecore e dei bovi; Rendic. Accad. Scienz. institut. Bologna, 1880-81. p. 123-130.

('81b.)—Sull' ovulatzione dei distomi epatico e lanceolato delle pecore e dei buoi; Bull. d. sc. 6. s. VII. 443-447. med. di Bologna.

('80c.)—Dell 'adattamento della specie all 'ambiente. Nuove ricerche sulla storia genetica dei Trematodi. Mem. dell' Accad. d. sc. dell 'institut. di Bologna, 4 ser. II. p. 239.

('82.)—De l'adaptation des espéces au milieu ambiant., nouvelles recherches sur l'origine des Trematodes; Arch. Ital. de Biol. I. p. 439.

FLORANCE—

('66.)—La distomatose chez l'homme et les animaux. Thése de Strasbourg.

FOGLIATA—

('87.)—Esperienze per la cura della cachessia acquosa o distomiasi epatica; Giorn. Anat. Fisiol. e Patol. degli animali. Lo spallanzani; anno XVI. p. 61.

FRANCIS, M.—

'91.—Liver Flukes; Texas Agric. Expm. Sta. Bullet. No. XVIII. Oct., 9 pgs. 6 orig. fig. Rev. in C. f. B. u. P., 1892. Bd. No. 19, p. 608.

FRIEDBERGER—

('78.)—Zur Kenntniss d. Egelseuch d. Schafe; Dent. Zeit. f. Thier. 4 Bd. p. 145-166.

FRÖLICH—

('02.)—Der Naturforscher XXIX., p. 5-96.

FROMAGE de FEUGRÉ.—

('10 )—Correspondence I. Paris. Vide Grognier I, 10.

GAEDE—

('17.)—*Distoma hepaticum*; Diss. de insect. Ver. struct. Kiliæ, p. 8.

GERLACH—

(54.)—Preuss. Mittheil.,

('54.)—Mag. f. Theirheilk.

'62.—Gerichtliche Thierheilkunde p. 511. Berlin.

GHOSE, B. M.—

('69.)—A case of worms, distoma hepaticum, or liver fluke, in the human intestines; Indian. M. Gaz. Calcutta, IV. 210. (D. crassum?)

GRALL—

('87.)--Deux observations de douve chez l'homme; Arch. de méd. nav. Paris, XLVIII. 459-470.

GRIFFITH, J W. & HENFREY, A.—

'83.—*Distoma hepaticum*; The Micrographic Dictionary. London, p. 268.

GULLIVER—

('40.)—Notes on the Ova of *Distoma hepaticum* and on certain corpuscles obtained from the genus cysticercus; Proc. Zool. Soc. March. Tom. VIII. p. 30-31.

('41.)—Ann' Nat. Hist. Vol VI.
('42.)—Month. Journ. Med. Sci. Vol. II.
('42.)—Micros. Journ. & Struct. Rec. p. 95.
('42.)—Wiegmann's Arch. I. p. 298.

GURLT, E. F.—
('49.)—Lehrbuch der pathol. Anat. (Nachtrag) p. 120.

HAHN. L. et LEFÉVRE, Ed.—

('84.)—Douves; Dict. encyclop. des sc. méd. Paris. 1. s., XXX, 515-549.

HALDEMANN, S. S.—
'51.—Distoma (or Fasciola) hepaticum; Outlines of General Zoölogy.—N. Y., p. 46. pl. 77, fig. 36.

HALSTED, Byron—
('83.)—The Liver Fluke of Sheep. (Fasciola hepatica); Pop. Sci. Month. XXIII. 741.—Nature XXVI. 606.

HAMONT & FISCHER—
('34.)—De la cachexie aqueuse de l'homme et du mouton; Journ. de méd. Vétér. Théor. et Prat. p. 129.
'34.—The Rot in Sheep; The Veterinarian, Vol. VII. p. 537-546, 587-592. (Translation.)

HARLEY & BROWN—
'76.—*F. hepatica*; Histological Demonstration. London, p. 255-256. fig. 208-209.

HARNES, Carsten—
('91.)—Zur Aufnahme der Leberegelbrut; Berliner thierärzt. Wochenschr. Jahr. 7. No. 27. S. 249-250.

HARRISON, E.—
('04.)—Inquiry into Rot in Sheep and other animals.

HARROP, E. D.—
('70.)—Remarks on the Fluke (*F. hepatica*). Rep. Roy. Soc. Tasmania (1869), 1870, p. 12-16.

HASSALL, Albert—
'94a.—List of the Chief Epizoötics of Fascioliasis (Distomatosis);The Journ. of Comp. Med. and Vet. Arch. pp. 162-167.
'94b.--Bibliography of *F. hepatica* (the present paper).

HEDLEY—
('81.)—*Distomum hepaticum;* Veterinarian, p. 374.

HELLER, A.—
('78.)—Die Schmarozer der Leber; Handb. d. spec. Path. (Ziemssen). Leipz., VIII. 1. Hlft., 1. Abth. 427-440.—Trans. Cycl. Pract. M. (Ziemssen) N. Y. 1880, IX. 481-495.

HODGSON, J. T.—
'38.—On Specific Fever of Horses, Oxen, &c.; The Veterinarian. Vol. XI. p. 533.

HOEVEN, J. Van der—
'59.—*Distoma hepaticum;* Handboek der Dierkunde Erste Deel. Amsterdam. p. 211.

HUMBLE, Wm. Ed. & LUSH, Wm. V.—
('81.)—A case of *Distoma hepaticum* (liver fluke) in man; Brit. Med. Journ. II. p. 75.

HUTCHEON, D.—
'92.—Fluke in sheep; Agric. Journ. V. p. 141. (Cape Town.)
HUZARD, et TEISSIER—
('17.)—Instructions Sommaires, Paris.
JACKSON, W. H.—
('83.)—Note on the Life History of *Fasciola* (*Distoma*) *hepatica;* Zool. Anz. VI. pp. 248 250.
JAKSCH, V. R.—
('89.)—Klinisch Diagnost. im Krank. Wien. p. 186, f. 61.
JORDENS, Jo. Heinr.—
'02.—Entomologie und Helminthologie des Mensch. Körp Vol II. p. 64-66. pl. VII. fig. 13-14.
JOSEPH, G.—
('83.)—Vorläufige Mittheilung über die Jungendzustände des Leberegels; Zool. Anz. VI. p. 322.
JOY, T.—
'46.—On the Rot in Sheep; The Veterinarian. Vol. XIX. pp. 297–298.
KARKEEK, W. F.—
'31.—Notes on the Rot, (or iles of the Cornish Graziers); Veterinarian, Vol. IV., p. 573.
KING, E.—
'36.—On the propagation of Rot (by means of the eggs of *F. hepatica*) in Sheep; Veterinarian. Vol. IX. pp. 95–101.
KINGSLEY, John Sterling—
'85.—The Standard Natural History, Vol. I. The Lower invertebrates. Distoma hepaticum pp. 191–194. Fig. 168–174.
KITAZATO, Shibazaburo—
('84.)—(*Distoma hepaticum*); Chiuga Iji Shinpo, Tokei, No. 99, May 10.
KRIWONOGOW—
('86.)—Archiv. vétér. de Saint-Pétérbourg.
KUCHENMEISTER, Fred.—
'57.—On Animal & Vegetable Parasites of the Human Body—Vol. 1. Trans. by Edwin Lankester—Lond. 1857. D. hepaticum, pp. 247–272. Pl. V. fig. 1-10.
et ZURN—
('81.)—Paras. d. Mensch. 2 edit. Leipsig. p. 290. tav. VII. fig. 1. 2. 4. tav. VIII. fig. 14.
LAMARK—
('18.)—Anim. sans verteb III. 182. 2nd. Edit. III. 618.
LAMOUROUX—
'24.—Le distome hépatique; Dict. classique D'hist. nat. Vol. V. p. 563. & p. 608.
De LANESSAN, J L.—
'82.—'Distomum hepaticum; Manuel D'Hist. Nat. Méd. Paris, pp. 221–238, figs. 193–203.
LANKESTER, Edw.—
'57.—Appendix B. in a Manual of Animal & Vegetable Parasites of the Human body by Küchenmeister, pp. 433–437.
LEIDY, J.—
'73.—On *Distoma hepaticum;* Proc. Acad. Nat. Sci. Phila. pp. 364–365.

LEUNIS—
'86.—*Distomum hepaticum;* Synopsis der Zoologie. II. pp. 852–853. figs. 809–803–805, Hannover.
LESSONA—
('12.)—Les maladies des moutons Turin.
LEUCKART, R.—
('63.)—Die Menschl. Paras. Leipsig.
('81.)—Zool. Anz. N. 99. pp. 641–646.
('82a.)—Zool. Anz. V. pp. 524–528, 9. Oct.
('82b.)—Life History of the Liver Fluke; Journ. Roy. Micros. Soc. Lond. 2 ser. Vol. 2. pp. 342 344.
('82c.)—Z. Entwick. d. Leberegels (*Distoma hepaticum*) M. Taf. 8. S. A.; Arch. f. Naturg. 48 Jahr. 1 Bd. p. 80, 119. Taf. VIII.
('82d.) Sur le dévelopement de la douve de foie (*Distomum hepaticum*); Arch. Zool. Experm. T IO. 2. note p. XXV. XXVIII.
('83a-id.)—2 ser. T. L. N. 2. 1883, p XXV. XXVI.
('83b.)—Arch. des Sc. Physiques et nat. VIII. pp. 467–472.
'86.—The Parasites of Man. Edinb.
'89.—Die Paras d. Mensch. 2 Aufl. 1. Band. 4 Liefer. p. 179–328. f. 96–144.
LINDQUIST—
('82.)—*Distomum hepaticum;* Sidekrift Vet. Med. p. 180.
LINNÉ, Sir Charles—
'01.—A general system of nature through the three grand Kingdoms of Amimals, Vegetables & Minerals, &c. Translated from Gemelin's last Edition of the Celebrated Systema Naturæ, by Sir Charles Linne. By William Turton, M.D. IV Vols. 1800–1801. pp. 33–34.
LINSTOW, O. V.—
('75.)—Beobachtungen an neven und bekannten Helminthen; Arch. f. Naturg. I. p. 183.
('83.)—Arch. f. Naturg. XLIX, 308.
('86.)—Viaggi Fedtschenko. Vermi (lavoro russo) Mosca, p. 31, figs. 51–52.
LITTLEWOOD, W.—
('87.)—*Distomum hepaticum;* Veterinarian, Aug. p. 546.
LUCET—
('90 )—Distomatose du poumon (Vache); Rec. de Méd. Vétér., p. 548.

(*To be continued.*)

